# Association Between Healthcare Accessibility Barriers and Perceived Healthcare Fairness Among Chinese Residents: An Empirical Study Based on CSS 2023 Data

**DOI:** 10.3390/healthcare14213628

**Published:** 2026-10-26

**Authors:** Hao Wei, Yisong Feng, Shuangjiang Zheng, Hao Wu

**Affiliations:** 1 School of Public Health, Chongqing Medical University, Chongqing 400016, China; 2 Key Laboratory of Digital Health and Intelligent Medicine, Chongqing Municipal Health Commission, Chongqing 400016, China; 3 Center for Big Data and Intelligent Medicine, The First Affiliated Hospital of Chongqing Medical University, Chongqing 400016, China; 4 Chongqing Translational Medicine Center, Chongqing 400016, China

**Keywords:** healthcare accessibility barriers, perceived healthcare fairness, medical insurance, exploratory associational path analysis, Chinese residents, CSS

## Abstract

**Objective**: Barriers to healthcare accessibility and individuals’ perceived fairness of medical services are crucial indicators for evaluating the performance of a health system. An in-depth analysis of the statistical associations among these factors and the role played by different types of medical insurance can provide targeted, evidence-based references for policy formulation in China. **Methods**: Data were analyzed from 9109 respondents of the 2023 Chinese Social Survey (CSS2023). Descriptive statistics, Spearman correlation analysis, multinomial logistic regression, and path analysis based on structural equation modeling were employed to examine the distribution of barriers and perceived fairness, the correlations among them, the differential impacts of various obstacles on perceived fairness, and the statistical pathways between insurance types and perceived fairness. **Results**: Economic barriers (reported as severe by 48.16% of respondents) and capacity barriers (reported as severe by 22.48%) were the most prevalent types of obstacles. All five types of barriers exhibited significant negative correlations with perceived healthcare fairness (*p* < 0.01), with economic barriers (ρ = −0.309) and capacity barriers (ρ = −0.267) demonstrating the strongest associations. Multinomial logistic regression revealed a progressively smaller magnitude of association across the outcome contrasts: for each one-unit increase in the economic-barrier score, the odds ratios for reporting “very unfair,” “somewhat unfair,” and “fair” (compared to “very fair”) were 2.30, 1.77, and 1.23, respectively (all *p* < 0.001), while the impact of geographical barriers was comparatively limited. Lower levels of perceived fairness were reported by adults aged 31–45 years and 46–60 years, divorced individuals, and the unemployed. Path analysis indicated that Urban Employee Basic Medical Insurance (indirect statistical association = 0.022, 95% CI: 0.002–0.042, *p* = 0.029) and Urban Resident Basic Medical Insurance (indirect statistical association =0.050, 95% CI: 0.001–0.099, *p* = 0.044) were significantly associated with higher perceived fairness, and a portion of these associations was statistically consistent with the reduction in accessibility barriers, whereas no statistically significant indirect associations were detected for Government-funded Medical Insurance, NRCMS, or Critical Illness Insurance. Given the small sample sizes for Government-funded Medical Insurance (n = 189) and Critical Illness Insurance (n = 83), these non-significant findings should not be interpreted as evidence of the absence of an association. **Conclusions**: Economic burdens and substandard medical services are the primary drivers of perceived inequity. Urban employee and resident insurance programs were statistically associated with higher perceived fairness, and a portion of these associations was consistent with the reduction in accessibility barriers, whereas rural and critical illness schemes lack such an indirect association. Policy priorities should focus on alleviating financial burdens, improving healthcare quality, strengthening insurance schemes for vulnerable groups, and addressing the specific needs of adults aged 31–45 years and 46–60 years, divorced, and unemployed populations to advance healthcare fairness in China.

## 1. Introduction

Against the backdrop of the ongoing advancement of Universal Health Coverage (UHC) and the implementation of the Healthy China strategy, public demand for healthcare services has evolved from merely “accessing medical care” to seeking “high-quality and equitable care” [1]. In response, the Chinese government has successively issued policy documents such as the Healthy China 2030 Plan and the Notice on Launching Themed Activities to Improve the Medical Experience and Enhance Patient Satisfaction, which explicitly mandate the need to address the urgent concerns of the population in accessing healthcare, and improving the accessibility and fairness of medical services [2,3]. However, inequities in the geographic and economic distribution of medical resources, alongside significant disparities in management systems across hospitals, constitute systemic barriers to accessibility [4]. These barriers leave certain population segments facing various obstacles during their medical journeys, profoundly impacting their care experience and satisfaction levels [3,5,6]. For vulnerable groups in particular, the convergence of economic, geographic, institutional, and sociocultural obstacles is more likely to result in compounded adverse effects [7,8,9].

Barriers to healthcare accessibility and individuals’ subjective perceptions of healthcare fairness are critical indicators for evaluating the performance of a health system [10]. Among the numerous obstacles, health insurance functions not only as a core instrument for mitigating economic barriers but may also itself become a component of institutional impediments to care [11]. Therefore, a thorough understanding of the intrinsic relationship between accessibility barriers and public perceptions of healthcare fairness, as well as the role of insurance within this statistical association, can provide targeted empirical evidence and policy-relevant insights for optimizing China’s medical security system design and accurately identifying intervention targets.

Although existing literature has preliminarily established associations between medical barriers and health outcomes, most current research focuses on measuring objective health inequalities. Systematic analyses of the characteristics and pathways linking multidimensional healthcare accessibility barriers to public perceptions of healthcare fairness, from the perspective of patients’ subjective experiences, remain comparatively scarce [12,13]. Consequently, there is a notable lack of comprehensive empirical investigation into the distributional characteristics of barriers encountered by Chinese residents during their healthcare-seeking process, their subjective perceptions of healthcare fairness, and how the medical insurance system may be statistically associated with this relationship. Based on the above analysis, this study proposes the following exploratory hypotheses: H1: The five types of healthcare accessibility barriers (geographic, appointment, waiting time, economic, and capacity) are significantly negatively correlated with perceived healthcare fairness. H2: Healthcare accessibility barriers may play a pathway role in the association between different types of medical insurance and perceived healthcare fairness, with the strength of these associations varying across insurance types. It should be noted that the data used in this study are cross-sectional, which precludes establishing the temporal sequence of the variables. Therefore, the above hypotheses aim to explore statistical association patterns among the variables rather than to test strict causal mediation relationships. The findings will provide an empirical basis for hypothesis generation in future longitudinal follow-up or instrumental variable studies. To this end, using data from the 2023 Chinese Social Survey (CSS 2023, N = 9109), this study employs descriptive statistics, Spearman correlation analysis, multinomial logistic regression, and path analysis based on structural equation modeling to systematically examine the characteristics of the associations between healthcare accessibility barriers and perceived fairness, and to explore the potential pathways through which insurance types may function in this relationship.

## 2. Methods

### 2.1. Study Design and Sample

Data for this study were derived from the Chinese Social Survey (CSS), a major project of the Chinese Academy of Social Sciences. The CSS is a probability-based household survey initiated in 2005 by the Institute of Sociology, Chinese Academy of Social Sciences, and is conducted biennially. CSS 2023, the ninth wave of this series, focuses on the theme of “Social Change and Chinese-style Modernization,” encompassing multidimensional content including family structure, employment status, economic conditions, living standards, social security, social values, social evaluation, social participation, political engagement, and voluntary services. This survey covered 624 villages/neighborhood committees across 31 provinces, autonomous regions, and municipalities directly under the central government, collecting a total of 13,035 valid questionnaires [14]. Although the CSS employs a stratified multistage probability sampling design, the analytical dataset available to us did not include sampling weights, stratification, or cluster variables. Therefore, standard errors in the path analysis were not adjusted for the complex survey design, and the bootstrap procedure was conducted without incorporating survey weights. We acknowledge that this may affect the precision of the estimates and the validity of the confidence intervals, and this limitation is discussed in the Limitations section.

**Figure 1:**
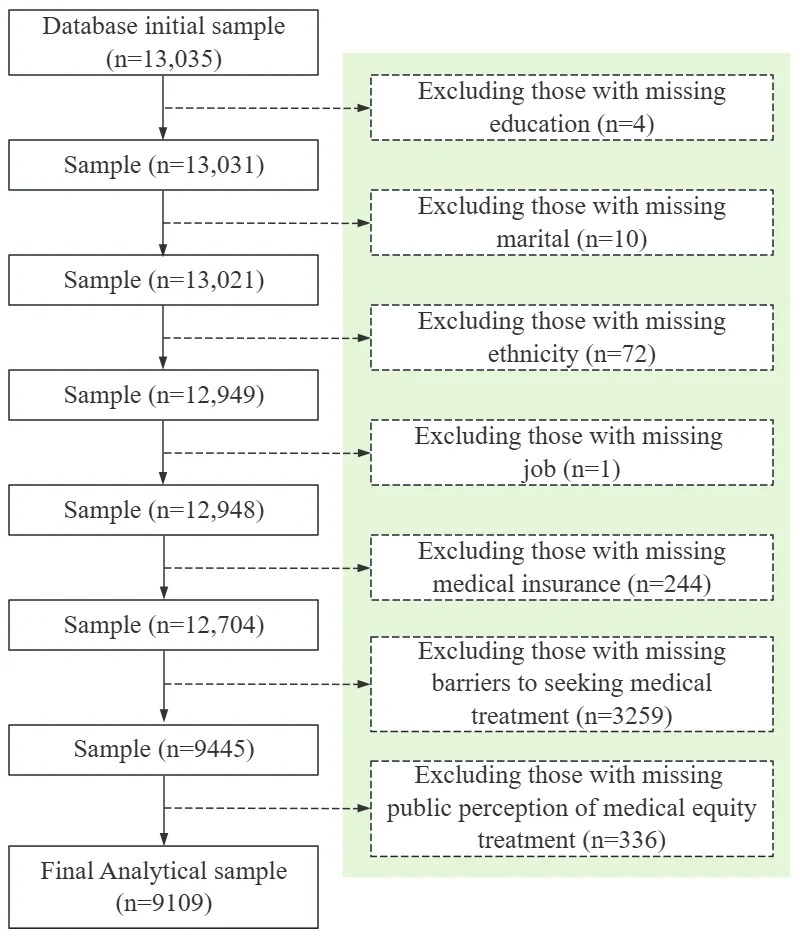
Sample screening flowchart.

Following the definition of core variables, strict exclusion criteria were applied; complete-case analysis was used, and the final analysis included only participants with complete data on all core variables. As shown in Figure 1, after screening, the final analytic sample comprised 9109 respondents (69.9% of the original 13,035 respondents). Table 1 presents the basic demographic characteristics of the sample. A comparison of the sociodemographic characteristics between included and excluded respondents is presented in Table S1. It should be noted that the total number of excluded respondents was 3926, but some excluded respondents also had missing data on sociodemographic variables, so the column totals vary across variables. The data used in this study were downloaded from the official CSS website (http://csqr.cass.cn, accessed 1 May 2026).

For variable processing in this study, age was categorized into four groups: 17–30, 31–45, 46–60, and 61–75 years. Educational attainment was collapsed into three levels based on academic qualifications: low (illiterate and primary school), middle (junior high school, senior high school, secondary specialized school, vocational high school, and technical school), and high (associate degree, bachelor’s degree, and postgraduate degree). Marital status was reclassified from the original questionnaire into four categories: unmarried (including cohabiting), married (including first marriage and remarried with spouse), divorced, and widowed. Ethnicity was simplified into two groups: Han and other ethnic minorities. Employment status was classified into four types: employed, employed but currently on leave, unemployed, and students. Type of work was further categorized into three groups: agricultural work (including exclusively farming, primarily non-agricultural with part-time farming, and primarily farming with part-time non-agricultural work), non-agricultural work, and no work. Health insurance coverage was dichotomized into insured and uninsured; among those insured, further subcategories included Urban Employee Basic Medical Insurance (UEBMI), Urban Resident Basic Medical Insurance (URBMI), Government-funded Medical Insurance, New Rural Cooperative Medical Scheme (NRCMS), and Critical Illness Insurance. Based on the actual protection capacity of China’s healthcare security system, respondents with multiple insurance types were uniquely classified according to a priority scheme: Government-funded Medical Insurance > UEBMI > URBMI > NRCMS > Critical Illness Insurance > No Insurance. The hierarchy was selected based on the principle of “primary coverage,” reflecting the actual reimbursement priority and coverage level of each insurance scheme in China’s healthcare system. This ordering is consistent with the institutional design of China’s medical insurance system. Specifically, government-funded medical insurance and UEBMI are primary insurance schemes with independent enrollment and contribution structures, whereas URBMI and NRCMS are also primary schemes but with lower contribution levels. Critical Illness Insurance, by contrast, is a supplementary scheme that operates on top of basic insurance and cannot be held independently. This classification is consistent with the institutional design of China’s medical insurance system.

For healthcare accessibility barriers, five core questions were included: excessive distance to the hospital, long waiting times for medical or surgical appointments, long waiting times in queues for consultations, excessive medical costs, and low medical skill levels. These five dimensions were conceptually derived from the accessibility framework proposed by Penchansky and Thomas (1981), which defines access as the degree of fit between patients and the healthcare system across dimensions of availability, accessibility, accommodation, affordability, and acceptability [15]. Following Frenk (1985), we distinguish accessibility (the potential for obtaining care) from access (the actual utilization of services); the five barrier items in this study capture accessibility rather than actual utilization [16]. Each barrier was scored from 0 to 3, representing severity levels ranging from “no problem” to “very severe,” with higher scores indicating more severe barriers. Perceived fairness of public healthcare was categorized into four levels ranging from “very unfair” to “very fair,” also scored from 0 to 3, with higher scores indicating a higher level of perceived fairness. The dimensionality of the five barrier items was examined using exploratory factor analysis (EFA) with polychoric correlations and confirmatory factor analysis (CFA). The EFA and CFA were conducted on the same sample of 9109 participants; therefore, the CFA should be interpreted as a confirmatory check on the structure identified in the EFA rather than as an independent validation. The EFA revealed a single-factor solution with an eigenvalue of 2.73, explaining 54.1% of the variance, and all factor loadings exceeded 0.54 (range: 0.54–0.90). The one-factor CFA showed acceptable fit (CFI = 0.992, TLI = 0.985, SRMR = 0.048), and a two-factor model showed improved fit (CFI = 0.999, TLI = 0.997, RMSEA = 0.047, SRMR = 0.022), with a high correlation between the two factors (r = 0.792). Although the two-factor model showed better fit, we retained the single composite score for the following reasons. First, the two factors were highly correlated (r = 0.792), indicating that they share substantial common variance and likely reflect a single higher-order construct of perceived difficulty in accessing care. Second, the one-factor model demonstrated acceptable fit (CFI = 0.992, TLI = 0.985, SRMR = 0.048), supporting the use of a general barrier factor. Third, the composite score was used only in the path analysis as a parsimonious summary measure, while the primary analysis (multinomial logistic regression) examined each barrier separately, thereby preserving the distinct contributions of each barrier domain. Fourth, using a single composite score is consistent with our theoretical framework, which conceptualizes the five barriers as manifestations of a common underlying construct of healthcare accessibility [15]. The composite barrier score was calculated as the mean of the five items, which preserves the original 0–3 metric, facilitates interpretation, and is robust to missing items (at least four of five items were required to be non-missing). Cronbach’s α for the five items was 0.79.

### 2.2. Statistical Analysis

R statistical software version 4.4.1 (R Foundation) was used for data analysis. Frequencies and percentages were employed to describe the distribution of sociodemographic characteristics and core variables. Spearman’s rank correlation analysis was performed to examine the strength and direction of associations among the five types of healthcare accessibility barriers and between each barrier and perceived healthcare fairness.

Given that perceived healthcare fairness is an ordinal variable with four categories (“very unfair,” “somewhat unfair,” “relatively fair,” and “very fair”), we first considered an ordinal logistic regression (proportional-odds model) as the primary analytical approach. However, the proportional-odds assumption was violated, as indicated by the test of parallel lines (χ^2^ = 241.617, df = 40, *p* < 0.01), suggesting that the effects of the healthcare accessibility barriers differed across the thresholds of perceived fairness. We therefore used multinomial logistic regression as the primary model, which does not require the proportional-odds assumption and allows the effects of predictors to vary across outcome categories. Multinomial logistic regression models were used to test the effects of different types of healthcare accessibility barriers on perceived healthcare fairness. Perceived healthcare fairness served as the dependent variable (reference group: “very fair”), with the five healthcare accessibility barriers as the core independent variables, while controlling for sex, age group, educational attainment, marital status, ethnicity, employment status, type of work, and insurance type as covariates. Regression results were presented as odds ratios (ORs) with 95% confidence intervals (CIs). It should be noted that the odds ratios reported in the multinomial logistic regression compare different levels of the outcome (e.g., “very unfair” vs. “very fair,” or “somewhat unfair” vs. “very fair”), rather than representing the effect of increasing levels of barrier severity.

Multicollinearity among the five barriers was assessed using variance inflation factors (VIFs) from a linear regression model with all five barriers entered simultaneously. The VIFs ranged from 1.31 to 2.14, with the highest values observed for appointment barriers (VIF = 2.14) and waiting-time barriers (VIF = 2.14), and all values were below the conventional threshold of 5, indicating acceptable multicollinearity. Sensitivity analyses using individual barrier models, the sum score, and the first principal component were also conducted, and the results are reported in the Section 3.

To explore the pathways of association among insurance type, healthcare accessibility barriers, and perceived healthcare fairness, this study employed path analysis using structural equation modeling (SEM) with the lavaan package (version 0.6-21) in R (version 4.4.1). The path model was specified based on the theoretical framework linking insurance type to perceived fairness through healthcare accessibility barriers. The model included: (a) a path from insurance type to the composite barrier score (path a); (b) a path from the composite barrier score to perceived fairness (path b); and (c) a direct path from insurance type to perceived fairness (path c’). Adjustment variables were included in both the mediator and outcome equations. The same set of adjustment variables as in the multinomial logistic regression was included in both the mediator and outcome equations: sex, age group, educational attainment, marital status, ethnicity, employment status, and type of work. These variables were selected based on their established associations with healthcare access and perceived fairness in the previous literature. Given the cross-sectional nature of the survey data, which precludes establishing temporal causal order among the variables, all estimated results are interpreted as statistical associations rather than causal mediation effects [17]. The dependent variable (perceived healthcare fairness) was a four-category ordinal variable, for which the Weighted Least Squares Mean and Variance adjusted (WLSMV) estimator, appropriate for ordinal data, was used. The mediator variable (composite score of healthcare accessibility barriers) was derived by taking the mean of five ordinal items (each scored 0–3) and was treated as a continuous variable in the model. This treatment was justified by the high internal consistency among the barrier items (Cronbach’s α = 0.79). The model was just-identified (df = 0), meaning that the number of free parameters equaled the number of data points. In such models, fit indices such as CFI, RMSEA, and SRMR are not informative and should not be used to evaluate model fit. We therefore did not apply generic fit thresholds; instead, model evaluation focused on the theoretical plausibility and statistical significance of the path coefficients. The significance of the indirect statistical association (i.e., path a × path b) was assessed using bias-corrected bootstrap methods (with 2000 resamples) to construct 95% confidence intervals; a statistical association was considered significant if the confidence interval did not include zero. All 2000 bootstrap replications converged successfully. The CSS 2023 dataset used in this study did not include sampling weights, stratification, or cluster variables. Therefore, standard errors in the path analysis were not adjusted for the complex survey design, and the bootstrap procedure was conducted without incorporating survey weights. We acknowledge that this may affect the precision of the estimates and the validity of the confidence intervals, and this limitation is discussed in the Limitations section. This is acknowledged as a limitation in the Limitations section. Due to the inherent limitations of cross-sectional data, all path analysis results in this study should be interpreted as exploratory evidence of association.

## 3. Results

### 3.1. Sociodemographic Characteristics of the Study Population

Table 1 presents the sociodemographic characteristics of the sample included in this study. A total of 9109 valid cases were included. Regarding sex distribution, female respondents (5152/9109, 56.56%) outnumbered male respondents (3957/9109, 43.44%). In terms of age distribution, the 46–60 years age group constituted the largest proportion of respondents (3115/9109, 34.20%), followed by the 31–45 years group (2780/9109, 30.52%), with the 17–30 years (1635/9109, 17.95%) and 61–75 years (1579/9109, 17.33%) groups having comparable proportions. Regarding educational attainment, respondents with middle-level education comprised the largest proportion (4461/9109, 48.97%), followed by those with high-level (2572/9109, 28.24%) and low-level (2076/9109, 22.79%) education. For marital status, married respondents constituted the vast majority (6997/9109, 76.81%), while unmarried (1468/9109, 16.12%), divorced (332/9109, 3.64%), and widowed (312/9109, 3.43%) respondents accounted for smaller proportions. Regarding ethnicity, Han respondents were predominant (8331/9109, 91.46%). In terms of employment status, employed respondents accounted for the highest proportion (5850/9109, 64.22%), unemployed respondents accounted for 24.79% (2258/9109), and students (571/9109, 6.27%) and those employed but currently on leave (430/9109, 4.72%) accounted for smaller proportions. For type of work, non-agricultural workers (3559/9109, 39.07%) slightly outnumbered agricultural workers (2723/9109, 29.89%), while those without work accounted for 31.04% (2827/9109). Regarding insurance type, the New Rural Cooperative Medical Scheme was the most prevalent type among respondents (3938/9109, 43.23%), followed by Urban Employee Basic Medical Insurance (2465/9109, 27.06%); respondents without health insurance accounted for 12.08% (1100/9109), while Government-funded Medical Insurance (189/9109, 2.07%) and Critical Illness Insurance (83/9109, 0.91%) accounted for smaller proportions. Among the 9109 respondents, 1247 (13.7%) reported having multiple insurance types.

### 3.2. Distribution of Severity of Healthcare Accessibility Barriers

Table 2 presents the distribution of the five types of healthcare accessibility barriers. Overall, respondents reported markedly different levels of severity across barriers. Economic barriers (excessive medical costs) were the most severe type of barrier reported, with a combined proportion of respondents selecting “relatively severe” or “very severe” reaching 48.16% ((2673 + 1714)/9109). Waiting time barriers (excessive waiting time in queues for consultations) ranked second, with a combined proportion of 28.56% ((1790 + 812)/9109). In contrast, geographic barriers (excessive distance to the clinic/hospital) were the least reported, with only 16.62% ((1025 + 489)/9109) selecting “relatively severe” or “very severe”. Appointment barriers (excessive waiting time for medical or surgical appointments) had a severe proportion of 20.04% ((1273 + 552)/9109), and capacity barriers (low medical skill levels) had a severe proportion of 22.48% ((1444 + 604)/9109). These findings indicate that economic burden and waiting time are the most prominent barriers in the healthcare-seeking process represented in this sample, while geographic accessibility issues are relatively less severe.

### 3.3. Distribution of Perceived Healthcare Fairness

Table 3 presents the overall distribution of respondents’ evaluations of perceived healthcare fairness. The results indicated that respondents’ evaluations were generally positive. The proportion selecting “relatively fair” was highest, at 56.5% (5146/9109), while 18.8% (1708/9109) selected “very fair”. The proportion selecting “somewhat unfair” was 18.8% (1709/9109), while only 6.0% (546/9109) selected “very unfair”. Overall, 75.3% of respondents perceived public healthcare as “relatively fair” or “very fair”, suggesting that the current public healthcare system has gained recognition from the majority of the public regarding fairness. Nevertheless, approximately one-quarter of respondents (24.8%) held negative evaluations, indicating room for improvement in healthcare fairness.

### 3.4. Correlation Analysis of Healthcare Accessibility Barriers and Perceived Fairness

**Figure 2:**
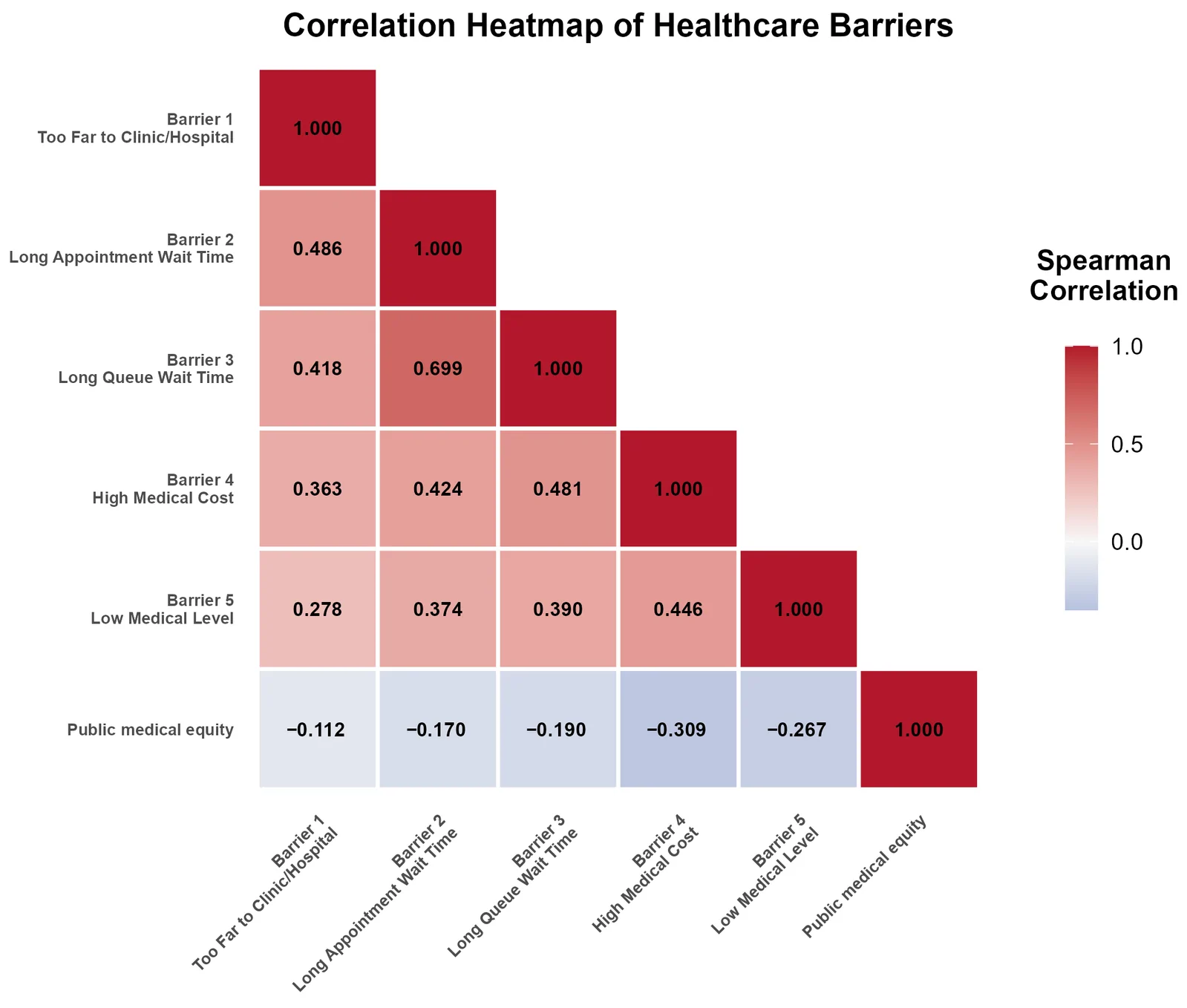
Spearman correlation heatmap between healthcare accessibility barriers and perceived healthcare fairness.

Figure 2 presents the Spearman correlation matrix among the five types of healthcare accessibility barriers and perceived healthcare fairness. The results showed that all five barriers were significantly negatively correlated with perceived healthcare fairness (*p* < 0.01). Among these, economic barriers (excessive medical costs) exhibited the largest absolute correlation coefficient (ρ = −0.309), indicating the strongest association with perceived fairness; capacity barriers (low medical skill levels, ρ = −0.267) ranked second; geographic barriers (ρ = −0.112) showed a relatively weaker correlation. These findings preliminarily suggest that alleviating economic burden may be a key entry point for enhancing public perceptions of healthcare fairness. Additionally, significant positive correlations were observed among the various barriers. The correlation between appointment barriers and waiting time barriers was strongest (ρ = 0.699), suggesting substantial overlap between these two time-related barriers and potential collinearity. Economic barriers showed moderate correlations with waiting time barriers (ρ = 0.481) and capacity barriers (ρ = 0.446), suggesting that “high costs,” “long waits,” and “low skill levels” are interconnected issues. This correlation pattern provides a preliminary basis for subsequent regression analyses.

### 3.5. Differential Effects of Various Healthcare Accessibility Barriers on Perceived Healthcare Fairness

**Figure 3:**
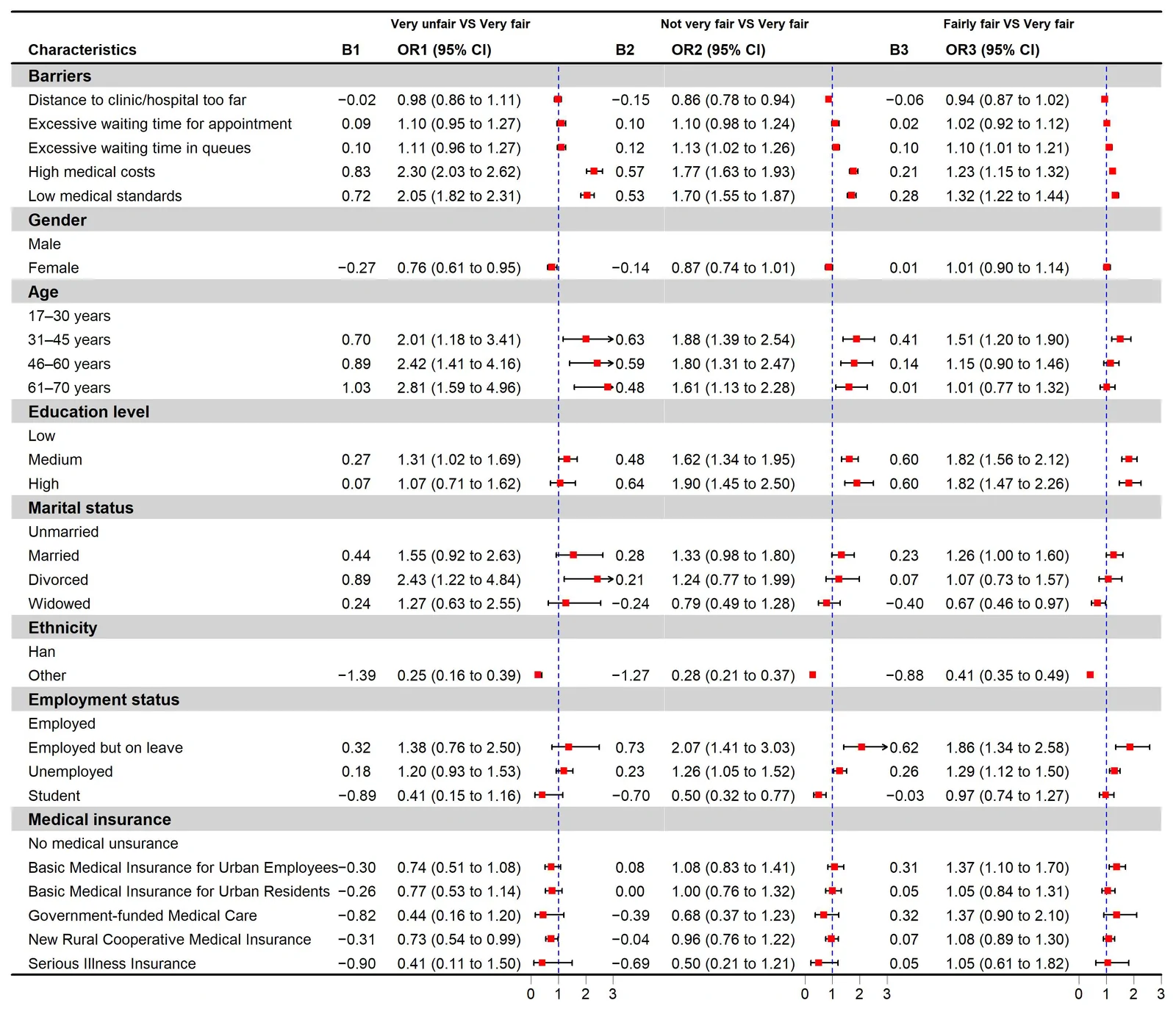
Results of multinomial logistic regression analysis: Associations between different types of healthcare accessibility barriers and perceived healthcare fairness. Each odds ratio corresponds to a one-unit increase in the barrier severity score (range: 0–3). The reference category for the dependent variable is “very fair.”.

Figure 3 presents the results of the multinomial logistic regression analysis examining the effects of the five types of healthcare accessibility barriers and control variables on perceived healthcare fairness. Due to high collinearity between the “employment status” and “type of work” variables, only the “employment status” variable was retained in the final multinomial logistic regression model to avoid parameter redundancy. Using “very fair” as the reference group, the five types of healthcare accessibility barriers were included as core independent variables, with sex, age, educational attainment, marital status, ethnicity, employment status, and insurance type included as covariates, to analyze the effects of each factor on different levels of perceived fairness.

Regarding healthcare accessibility barriers, economic barriers were a significant independent factor influencing perceived healthcare fairness. For each one-unit increase in the economic-barrier score, the odds of reporting “very unfair” versus “very fair” were 2.30 (95% CI: 2.03–2.62); the odds of reporting “somewhat unfair” versus “very fair” were 1.77 (95% CI: 1.63–1.93); and the odds of reporting “relatively fair” versus “very fair” were 1.23 (95% CI: 1.15–1.32). Capacity barriers were also significant, with ORs of 2.05 (95% CI: 1.82–2.31), 1.70 (95% CI: 1.55–1.87), and 1.32 (95% CI: 1.22–1.44), respectively, across the three comparisons. Geographic barriers showed a significant negative association, with an OR of 0.86 (95% CI: 0.78–0.94) in the “somewhat unfair vs. very fair” comparison. Waiting time barriers had ORs of 1.13 (95% CI: 1.02–1.26) and 1.10 (95% CI: 1.01–1.21) in the “somewhat unfair vs. very fair” and “relatively fair vs. very fair” comparisons, respectively. Appointment barriers did not reach statistical significance in any comparison.

Regarding demographic characteristics, age was a significant independent associated factor. Using the 17–30 years age group as the reference, the OR for the 31–45 years group in the “very unfair” comparison was 2.01 (95% CI: 1.18–3.41), and for the 46–60 years group, the OR was 2.42 (95% CI: 1.41–4.16). Regarding sex, using male as the reference, female respondents had a significant OR of 0.76 (95% CI: 0.61–0.95) in the “very unfair vs. very fair” comparison, indicating that women had lower odds of reporting “very unfair” than men; no significant sex differences were observed in the other two comparisons. For educational attainment, using low education as the reference, the ORs for middle education in the “somewhat unfair” and “relatively fair” comparisons were 1.62 (95% CI: 1.34–1.95) and 1.82 (95% CI: 1.56–2.12), respectively; the corresponding ORs for high education were 1.90 (95% CI: 1.45–2.50) and 1.82 (95% CI: 1.47–2.26). Regarding marital status, using unmarried as the reference, the OR for divorced individuals in the “very unfair” comparison was 2.43 (95% CI: 1.22–4.84). For ethnicity, using Han as the reference, the ORs for other ethnicities in the three comparisons were 0.25 (95% CI: 0.16–0.39), 0.28 (95% CI: 0.21–0.37), and 0.41 (95% CI: 0.35–0.49), respectively. For employment status, using employed as the reference, the ORs for those employed but currently on leave in the “somewhat unfair” and “relatively fair” comparisons were 2.07 (95% CI: 1.41–3.03) and 1.86 (95% CI: 1.34–2.58), respectively; the ORs for unemployed individuals in the corresponding comparisons were 1.26 (95% CI: 1.05–1.52) and 1.29 (95% CI: 1.12–1.50). For insurance type, using no insurance as the reference, the OR for NRCMS in the “very unfair” comparison was 0.73 (95% CI: 0.54–0.99); the OR for UEBMI in the “relatively fair” comparison was 1.37 (95% CI: 1.10–1.70).

To investigate the potential reasons for the counterintuitive OR of less than 1 for geographic barriers, we conducted multicollinearity diagnostics and sensitivity analyses. All VIFs were below 5 (range: 1.31–2.14), indicating that multicollinearity was not severe. When geographic barriers were entered alone into the multinomial logistic regression model (without the other four barriers), the OR for the “somewhat unfair vs. very fair” comparison was 1.36 (95% CI: 1.26–1.47, *p* < 0.001), consistent with the expected direction. When economic barriers were excluded from the model, the geographic-barrier OR attenuated toward 1.0 and became non-significant (OR = 0.94, 95% CI: 0.86–1.03). These results suggest that the counterintuitive effect of geographic barriers in the full model may reflect a suppression effect rather than severe multicollinearity, since the VIF for geographic barriers was low (1.33). The sensitivity analyses suggest that the inverse association observed in the fully adjusted model may reflect suppression arising from adjustment for correlated barriers; however, the underlying mechanism cannot be established from these data.

### 3.6. Path Analysis of Associations Among Insurance Type, Healthcare Accessibility Barriers, and Perceived Healthcare Fairness

**Figure 4:**
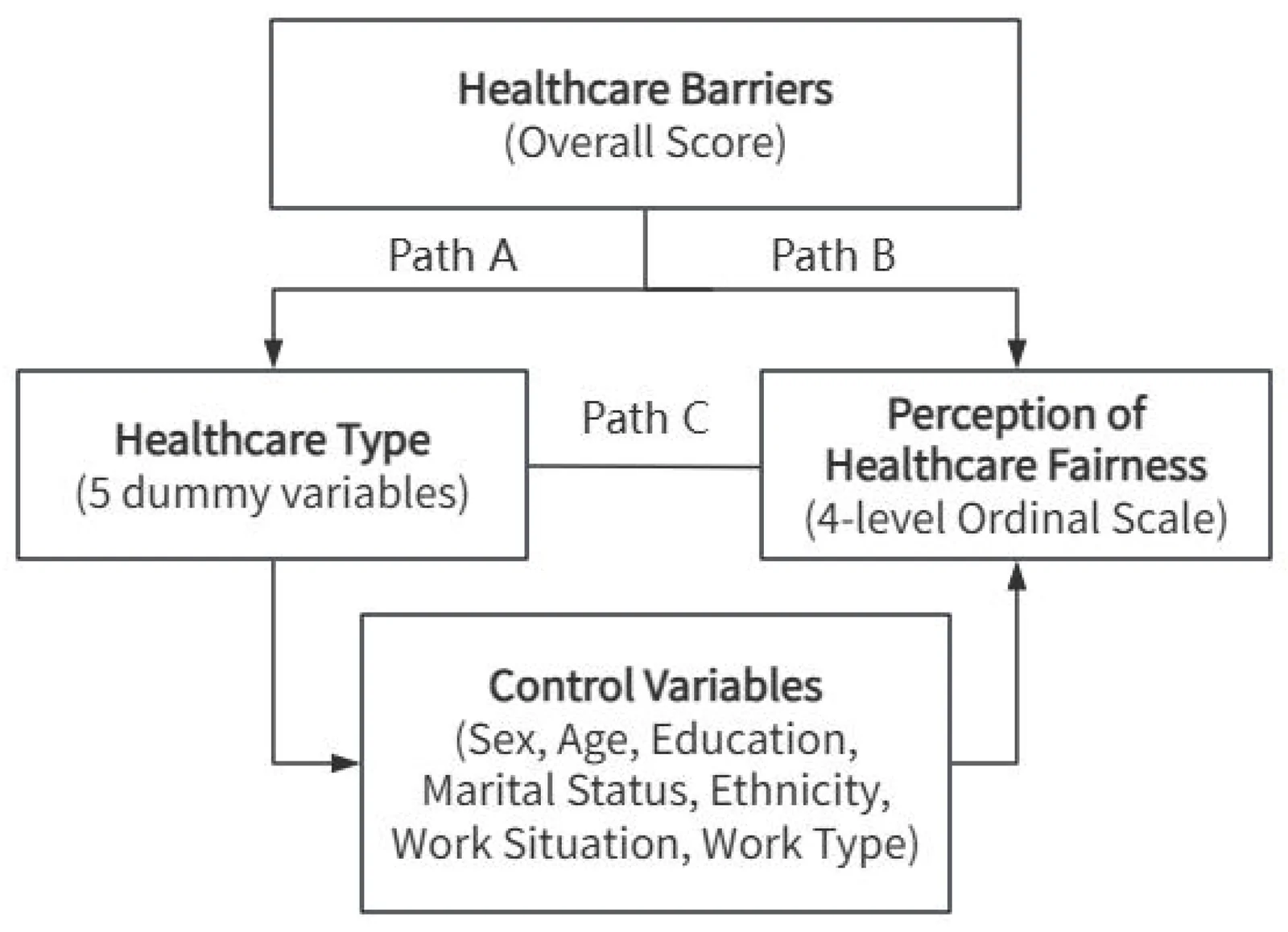
Path diagram.

In this section, path analysis based on structural equation modeling (SEM) was conducted to examine the statistical association patterns between five types of medical insurance and healthcare accessibility barriers as well as perceived fairness, using no insurance as the reference group. As shown in Figure 4, the path analysis involved three core pathways: Path a (insurance type → healthcare accessibility barriers): statistical association reflected the statistical association between different insurance types and healthcare accessibility barriers; Path b (healthcare accessibility barriers → perceived healthcare fairness) reflected the association between healthcare accessibility barriers and perceived fairness; and the indirect statistical association (a × b), the product of Path a and Path b, represented the indirect statistical association between insurance type and perceived fairness through healthcare accessibility barriers.

It should be noted that the path model in this study has zero degrees of freedom (df = 0), indicating a just-identified model, and the analysis was exploratory in nature; the cross-sectional design precludes causal inference, and all findings should be interpreted as statistical associations rather than causal effects. In this case, the fit indices (CFI = 1.000, RMSEA = 0.000, SRMR = 0.000) do not reflect model fit quality; rather, model evaluation should focus on the theoretical plausibility and practical significance of the estimated path coefficients [18].

The standardized coefficient for Path b (healthcare accessibility barriers → perceived fairness): statistical association was −0.455 (*p* < 0.001, 95% CI: −0.481, −0.429), indicating that more severe healthcare accessibility barriers were associated with lower perceived fairness, a finding highly consistent with the preceding correlation analysis results.

The indirect association effects varied across insurance types (Table 4). For Urban Employee Basic Medical Insurance (UEBMI), the standardized coefficient for Path a was −0.048 (*p* = 0.029, 95% CI: −0.092, −0.005), with an indirect statistical association of 0.022 (95% CI: 0.002–0.042, *p* = 0.029). For Urban Resident Basic Medical Insurance (URBMI), the Path a coefficient was −0.111 (*p* = 0.043, 95% CI: −0.218, −0.003), with an indirect statistical association of 0.050 (95% CI: 0.001–0.099, *p* = 0.044). These findings indicate that, for UEBMI and URBMI enrollees, there is a statistical association between reduced healthcare accessibility barriers and higher perceived fairness. In contrast, for Government-funded Medical Insurance (Path a = −0.008, *p* = 0.686; indirect statistical association = 0.004, *p* = 0.686), the New Rural Cooperative Medical Scheme (NRCMS; Path a = −0.013, *p* = 0.423; indirect statistical association = 0.006, *p* = 0.423), and Critical Illness Insurance (Path a = −0.034, *p* = 0.686; indirect statistical association = 0.016, *p* = 0.686), neither the Path a coefficients nor the indirect statistical association s reached statistical significance.

The above results indicate that, among UEBMI and URBMI enrollees, there exists a statistical association between reduced healthcare accessibility barriers and higher perceived fairness. However, it must be emphasized that, given the limitations of the cross-sectional data, the results of this study should be interpreted as exploratory evidence of association rather than causal inference.

It should be noted that Path b (barriers → fairness) was estimated as a pooled coefficient shared across all insurance-specific models because the mediator and outcome variables were identical. The differences across insurance types are therefore reflected in Path a (insurance → barriers) and the indirect effect (a × b), not in Path b.

## 4. Discussion

Based on national survey data from CSS 2023, this study systematically analyzed the relationship between healthcare accessibility barriers and perceived healthcare fairness among Chinese residents, and further explored the indirect statistical association between insurance type and perceived fairness via healthcare accessibility barriers. The results showed that all five types of healthcare accessibility barriers (geographic barriers, appointment barriers, waiting time barriers, economic barriers, and capacity barriers) were significantly negatively correlated with perceived healthcare fairness, a finding consistent with previous research [19,20,21]. Among these, economic barriers showed the strongest correlation with perceived fairness (ρ = −0.309), followed by capacity barriers (ρ = −0.267), while geographic barriers showed a relatively weaker correlation (ρ = −0.112). This indicates that, in the adjusted multinomial regression, economic barriers and capacity barriers were consistently associated with perceived fairness across all outcome contrasts, whereas appointment barriers were not statistically significant [22,23].

Examining the severity distribution of barriers, 48.16% of respondents reported economic barriers as “relatively severe” or “very severe,” 28.56% reported severe waiting time barriers, while only 16.62% reported severe geographic barriers. This pattern suggests that, in this sample, economic barriers were the most frequently reported severe barrier in this sample, followed by waiting-time barriers. It is important to distinguish between the prevalence of a barrier and the strength of its association with perceived fairness: while economic barriers were the most frequently reported, economic and capacity barriers showed the strongest adjusted associations with perceived fairness. This pattern resonates, to some extent, with the parallel policy orientations of “strengthening primary care and promoting equity” alongside “controlling costs and improving quality” that have been consistently advanced in China’s healthcare system reform in recent years [24].

### 4.1. Multilevel Effects of Healthcare Accessibility Barriers on Perceived Fairness

Multinomial logistic regression analysis further revealed hierarchical differences in the effects of various healthcare accessibility barriers on perceived fairness. For each one-unit increase in the economic-barrier score, the odds of reporting “very unfair” versus “very fair” were 2.30, and the odds of reporting “relatively fair” versus “very fair” were 1.23. Similar patterns were observed for capacity barriers. This finding indicates that economic and capacity barriers are strongly statistically associated with reduced perceived fairness, particularly with evaluations of “extreme unfairness.” One possible explanation is that high medical costs and low-quality medical services directly threaten patients’ basic health rights and interests, thereby more easily triggering strong perceptions of inequity [25].

In contrast, geographic barriers showed a significant negative association only in the comparison of “somewhat unfair vs. very fair” (OR = 0.86), while appointment barriers did not reach statistical significance in any comparison. This result may reflect, though cannot be confirmed by this study, the achievements of China’s recent efforts in medical infrastructure construction and the promotion of the tiered diagnosis and treatment system [26,27], whereby geographic distance and appointment waiting times are no longer primary sources of perceived healthcare inequity. It is worth noting that the association between geographical barriers and the sense of fairness in this study is relatively weak, but this does not mean that the problem of geographical accessibility has been solved [28]. Waiting time barriers remained significant at both the “somewhat unfair” and “relatively fair” levels, suggesting that waiting time issues remain a prevalent concern, albeit with a less intense impact on perceived fairness compared to economic barriers—a finding similar to existing research [21].

### 4.2. Differential Effects of Demographic Characteristics

This study found that age, educational attainment, marital status, ethnicity, employment status, and insurance type all significantly associated with perceived healthcare fairness. These findings are partially consistent with previous research while also revealing patterns specific to China [29].

Regarding age, using the 17–30 years age group as the reference, adults aged 31–45 years and 46–60 years had a significantly higher odds of reporting “very unfair” (ORs of 2.01 and 2.42, respectively). This finding is consistent with a cross-national study involving 11 high-income countries [30]. One possible explanation, though not directly tested in this study, is that older adults may have more favorable healthcare experiences and greater familiarity with the healthcare system. In contrast, adults aged 31–45 years and 46–60 years may face greater economic pressure and more intense work–life balance challenges, and may hold higher expectations regarding healthcare efficiency and quality, and are therefore more prone to perceptions of unfairness. This conclusion is similar to previous research [31].

Regarding educational attainment, compared to those with low education, middle- and high-education groups were more likely to report “somewhat unfair” and “relatively fair” rather than “very fair.” One possible explanation, though not directly tested here, is that higher educational attainment may be accompanied by improved health literacy and a better understanding of the healthcare system, enabling individuals to evaluate healthcare fairness more rationally rather than simply providing extreme assessments—a finding consistent with several studies [32,33]. This finding does not contradict previous research showing a positive correlation between educational attainment and healthcare service satisfaction, as “relatively fair” in this study still falls within the positive evaluation category [34].

Regarding marital status, divorced individuals had 2.43 times the odds of reporting “very unfair” compared to unmarried individuals, a finding consistent with existing research [35]. One possible explanation is that divorce may be associated with negative consequences including worsening economic conditions and diminished social support networks, factors that may amplify individuals’ perceptions of healthcare system inequities [36,37]. In contrast, widowed individuals had an OR of 0.67 in the comparison of “relatively fair vs. very fair,” suggesting a tendency to report “very fair.” One possible explanation is that this group may have greater reliance on the public healthcare system and a more appreciative attitude toward available services.

Regarding ethnicity, other ethnic groups had significantly lower odds of reporting unfairness across all levels of perceived fairness compared to the Han group (ORs ranging from 0.25 to 0.41). This finding requires cautious interpretation. One possible explanation, though not directly tested in this study, is that state preferential medical policies in ethnic minority regions may play a role; alternatively, differences in cultural values and expectation levels across ethnic groups may contribute [38]. It is worth noting that the sample size for the “other ethnic groups” category in this study was relatively small (778 individuals, 8.54%), which may affect the stability of the results.

Regarding employment status, groups “employed but currently on leave” and “unemployed” were more likely to report lower levels of perceived fairness compared to the “employed” group. One possible explanation is that those on leave may face the dual distress of work and health simultaneously, generating insecurity. They may worry about career development due to absenteeism while suffering physical and psychological stress from illness, a dual burden that may intensify this group’s perceptions of healthcare unfairness [39]. Unemployed groups (including both unemployed and retired individuals) have limited financial resources and greater sensitivity to medical costs, making them more susceptible to perceptions of unfairness.

Regarding insurance type, NRCMS enrollees had an OR of 0.73 in the “very unfair” comparison, indicating a lower odds of reporting extreme unfairness compared to those without insurance. This reflects the positive role of the New Rural Cooperative Medical Scheme in safeguarding the basic medical needs of rural residents [40]. UEBMI enrollees had an OR of 1.37 in the “relatively fair” comparison, suggesting a tendency to report moderate rather than extreme levels of fairness.

### 4.3. Path Analysis of the Association Between Insurance Type and Perceived Fairness: An Institutional Interpretation

The path analysis in this study revealed that Urban Employee Basic Medical Insurance (UEBMI; indirect statistical association = 0.022, *p* = 0.029) and Urban Resident Basic Medical Insurance (URBMI; indirect statistical association = 0.050, *p* = 0.044) were significantly associated with higher perceived fairness, whereas no such patterns were observed for Government-funded Medical Insurance, the New Rural Cooperative Medical Scheme (NRCMS), or Critical Illness Insurance. This is somewhat in line with the relevant studies [41].

This finding has important policy implications. One possible explanation is that the statistical association between UEBMI/URBMI and higher perceived fairness, which may be partly consistent with a ‘barrier reduction’ pathway, may be attributable to the design features of these two insurance schemes: they typically provide higher reimbursement rates, a broader choice of designated medical institutions, and more comprehensive outpatient pooling services, thereby being statistically associated with lower enrollees’ economic burden and uncertainty in the healthcare-seeking process. Consequently, these schemes exhibit a strong statistical “barrier–fairness” association. In contrast, NRCMS has relatively lower coverage levels and a limited capacity to alleviate healthcare accessibility barriers, failing to demonstrate a significant associational pathway. Critical Illness Insurance primarily targets major diseases and has a lesser impact on routine healthcare accessibility barriers, thus no significant indirect association was observed. The small sample sizes for Government-funded Medical Insurance (n = 189) and Critical Illness Insurance (n = 83) may have resulted in insufficient statistical power to detect significant indirect associations. Therefore, the non-significant indirect associations observed for these two schemes should not be interpreted as evidence that no association exists; rather, they indicate that the available data were insufficient to detect a statistically significant association. This finding complements existing research [40,42,43].

It must be emphasized that, given the cross-sectional design of this study, the above interpretations are inferences based solely on the statistical patterns observed in the data and should not be construed as evidence of causal mechanisms. Future research should employ longitudinal follow-up data or natural experiment designs (e.g., medical insurance policy reforms) to more rigorously examine the temporal relationships among insurance type, healthcare accessibility barriers, and perceived fairness.

### 4.4. Limitations and Future Directions

This study has several limitations. First, the cross-sectional design precludes causal inference regarding the relationships among variables. The association between healthcare accessibility barriers and perceived fairness may be subject to reverse causality or may be confounded by unmeasured confounders. Future research could employ longitudinal follow-up designs or instrumental variable methods to more rigorously test causal directions. Second, regarding variable measurement, healthcare accessibility barriers were assessed using self-reports of “the most recent healthcare experience,” which may be subject to recall bias and social desirability bias. Third, regarding sample representativeness, the final analytic sample had relatively higher proportions of individuals with high educational attainment (28.24%) and UEBMI enrollees (27.06%), which may limit the generalizability of the findings to populations with lower educational attainment and rural residents. Additionally, the small sample sizes for Government-funded Medical Insurance (n = 189) and Critical Illness Insurance (n = 83) resulted in insufficient statistical power. Consequently, the non-significant indirect associations observed for these two schemes should not be interpreted as evidence of the absence of an association; rather, they reflect limited statistical power to detect such associations. A comparison of included and excluded respondents (Table S1) showed that excluded respondents were more likely to be older, less educated, and without medical insurance. Because some excluded respondents also had missing data on sociodemographic variables, the comparison was based on available data for each variable. This suggests that the complete-case analysis may introduce some selection bias. Fourth, regarding variable control, this study was unable to include important factors that may be associated with perceived fairness, such as household income, self-rated health status, frequency of healthcare utilization, and perceived medical service quality; these omitted variables may lead to estimation bias. Future research should include as comprehensive a set of control variables as possible to improve model estimation accuracy. Fifth, all interpretations offered in the Discussion are post hoc and based on statistical associations observed in cross-sectional data; they should be regarded as hypothesis-generating and require further investigation.

Despite these limitations, this study makes important theoretical contributions and offers practical implications. Theoretically, to our knowledge, few studies have systematically examined the graded associations between five types of healthcare accessibility barriers and perceived healthcare fairness, revealing the central roles of economic and capacity barriers and validating the indirect statistical association through which insurance type influences perceived fairness via healthcare accessibility barriers [30]. This enriches the theoretical framework of healthcare fairness research and provides an analytical approach for future studies.

Practically, the findings of this study offer clear implications for health policy. First, reducing medical costs and improving medical quality may be potential areas for policy attention in efforts to enhance public perceptions of healthcare fairness. Governments may consider continuing to promote centralized drug procurement, healthcare payment reform, and medical quality regulatory system construction. Second, the findings identify adults aged 31–45 years and 46–60 years, divorced individuals, and unemployed groups as groups that may warrant particular policy attention with respect to perceived healthcare fairness, potentially through targeted economic assistance and psychological support. Furthermore, although no statistically significant indirect association was detected for NRCMS or Critical Illness Insurance in this study, the institutional design of these two schemes merits continued attention. Future research with larger and more representative samples is needed to determine whether these schemes are associated with healthcare accessibility barriers and perceived fairness.

## 5. Conclusions

In summary, all five types of healthcare accessibility barriers were significantly negatively correlated with perceived fairness. Economic barriers (excessive medical costs) and capacity barriers (low medical skill levels) were the most prominent influencing factors, exhibiting a progressively smaller magnitude of association across the outcome contrasts, wherein for each one-unit increase in barrier severity, the odds of reporting extreme unfairness increased. The exploratory associational path analysis revealed that Urban Employee Basic Medical Insurance (UEBMI) and Urban Resident Basic Medical Insurance (URBMI) exhibited significant positive statistical associations with higher perceived fairness, and a portion of these associations could be statistically associated with the reduction in healthcare accessibility barriers. In contrast, no statistically significant indirect associations were detected for Government-funded Medical Insurance, NRCMS, or Critical Illness Insurance. Therefore, reducing the burden of medical costs, improving medical service quality, and attending to the specific needs of vulnerable groups—including adults aged 31–45 years and 46–60 years, divorced individuals, and unemployed individuals—may be potential areas for policy attention in efforts to enhance public perceptions of healthcare fairness. Concurrently, further improving the institutional design of NRCMS and Critical Illness Insurance, and exploring factors associated with their relationship to perceived fairness, may represent potential directions for future healthcare reform.

## Figures and Tables

**Table 1:** Sociodemographic characteristics of the study population (n = 9109).

Characteristic	n	Percentage (%)
Sex		
Male	3957	43.44
Female	5152	56.56
Age		
17–30 years	1635	17.95
31–45 years	2780	30.52
46–60 years	3115	34.20
61–75 years	1579	17.33
Educational Attainment		
Low	2076	22.79
Middle	4461	48.97
High	2572	28.24
Marital Status		
Unmarried	1468	16.12
Married	6997	76.81
Divorced	332	3.64
Widowed	312	3.43
Ethnicity		
Han	8331	91.46
Other	778	8.54
Employment Status		
Employed	5850	64.22
Employed, currently on leave	430	4.72
Unemployed	2258	24.79
Student	571	6.27
Type of Work		
Agricultural work	2723	29.89
Non-agricultural work	3559	39.07
No work	2827	31.04
Insurance Type		
No insurance	1100	12.08
UEBMI	2465	27.06
URBMI	1334	14.64
Government-funded	189	2.07
NRCMS	3938	43.23
Critical Illness	83	0.91

Note: UEBMI = Urban Employee Basic Medical Insurance; URBMI = Urban Resident Basic Medical Insurance; NRCMS = New Rural Cooperative Medical Scheme.

**Table 2:** Distribution of severity of healthcare accessibility barriers (n = 9109).

Barrier Type	No Problem n (%)	Not Severe n (%)	Relatively Severe n (%)	Very Severe n (%)
1. Excessive distance to clinic/hospital	4775 (52.42)	2820 (30.96)	1025 (11.25)	489 (5.37)
2. Excessive waiting time for appointment	5036 (55.29)	2248 (24.68)	1273 (13.98)	552 (6.06)
3. Excessive waiting time in queue	4114 (45.16)	2393 (26.27)	1790 (19.65)	812 (8.91)
4. Excessive medical costs	2419 (26.56)	2303 (25.28)	2673 (29.34)	1714 (18.82)
5. Low medical skill levels	3990 (43.80)	3071 (33.71)	1444 (15.85)	604 (6.63)

**Table 3:** Distribution of perceived healthcare fairness (n = 9109).

Perceived Fairness	Frequency	Percentage	Cumulative Percentage
Very unfair	546	6.0	6.0
Somewhat unfair	1709	18.8	24.8
Relatively fair	5146	56.5	81.2
Very fair	1708	18.8	100.0
Total	9109	100.0	

**Table 4:** Path diagram of associations among insurance type, healthcare accessibility barriers, and perceived healthcare fairness

Insurance Type	Path a (X → M)	Path b (M → Y)	Indirect Statistical Association (a × b)	95% CI	*p*-Value
Government-funded	−0.008	−0.455 **	0.004	[−0.014, 0.021]	0.686
UEBMI	−0.048 *	−0.455 **	0.022	[0.002, 0.042]	0.029
URBMI	−0.111 *	−0.455 **	0.050	[0.001, 0.099]	0.044
NRCMS	−0.013	−0.455 **	0.006	[−0.009, 0.020]	0.423
Critical Illness	−0.034	−0.455 **	0.016	[−0.060, 0.091]	0.686

Path b represents a pooled estimate shared across all models because the mediator (composite barrier score) and outcome (perceived healthcare fairness) variables were identical across insurance-specific models; the differences across insurance types are reflected in Path a and the indirect effect (a × b). * *p* < 0.05, ** *p* < 0.001. CI = bias-corrected bootstrap 95% confidence interval. The results presented in this table are derived from path analysis based on cross-sectional data and reflect the strength of statistical associations; they do not represent causal mediation effects.

## Data Availability

Publicly available datasets were analyzed in this study. This data can be found here: http://csqr.cass.cn, accessed on 1 May 2026.

## Supplementary Materials

The following supporting information can be downloaded at: https://www.mdpi.com/article/10.3390/healthcare14213628/s1, Table S1: Comparison of sociodemographic characteristics between included and excluded re-spondents; Table S2: Multinomial logistic regression: Associations between healthcare accessibility barriers and perceived healthcare fair-ness (reference category: very fair); Table S3: Dimensionality analysis of the five healthcare-barrier items; Table S4: Sensitivity analysis of insurance classification.
