# Supplementary material for: Association Between Healthcare Accessibility Barriers and Perceived Healthcare Fairness Among Chinese Residents: An Empirical Study Based on CSS 2023 Data
Source: Healthcare (Basel). 2026 Oct 26;14(21):3628. doi: 10.3390/healthcare14213628 (PMC13762608; doi:10.3390/healthcare14213628)
Supplement: Supplementary file 1 [file healthcare-14-03628-s001.zip › healthcare-4526479-supplementary.pdf]

Table S1. Comparison of sociodemographic characteristics between included and excluded respondents.

Characteristic	Included (n = 9,109)	Excluded (n = 3,926)	p-value
Sex	n = 9109	n = 3926	<0.001
Male	3957	1850	
Female	5152	2076	
Age	n = 9109	n = 3926	<0.001
17-30 years	1635	393	
31-45 years	2780	877	
46-60 years	3115	1721	
61-75 years	1579	935	
Educational Attainment	n = 9109	n = 3922	<0.001
Low	2076	1461	
Middle	4461	1955	
High	2572	506	
Marital Status	n = 9109	n = 3916	<0.001
Unmarried	1468	430	
Married	6997	3099	
Divorced	332	195	
Widowed	312	192	
Ethnicity	n = 9109	n = 3854	<0.001
Han	8331	3442	
Other	778	412	
Employment Status	n = 9109	n = 3926	<0.001
Employed	5850	2619	
Employed currently on leave	430	152	
Unemployed	2258	1016	
Student	571	139	
Type of Work	n = 9109	n = 3905	<0.001
Agricultural work	2723	1598	
Non-agricultural work	3559	1174	
No work	2827	1133	
Insurance Type	n = 9109	n = 3838	<0.001
No insurance	1100	590	
UEBMI	2465	581	
URBMI	1334	487	
Government-funded	189	65	
NRCMS	3938	1996	
Critical Illness	83	119	

*Note: UEBMI=Urban Employee Basic Medical Insurance; URBMI=Urban Resident Basic Medical Insurance; NRCMS=New Rural Cooperative Medical Scheme.

Table S2. Multinomial logistic regression: Associations between healthcare accessibility barriers and perceived healthcare fairness (reference category: very fair).

Variable	Very unfair vs Very fair<br>OR (95% CI), p	Somewhat unfair vs Very fair<br>OR (95% CI), p	Relatively fair vs Very fair<br>OR (95% CI), p
**Healthcare accessibility barriers**			
Distance to clinic/hospital too far	0.98 (0.87–1.11), p = 0.753	0.86 (0.78–0.94), p = 0.001	0.94 (0.87–1.02), p = 0.140
Excessive waiting time for appointment	1.10 (0.95–1.27), p = 0.202	1.10 (0.98–1.24), p = 0.086	1.02 (0.92–1.12), p = 0.683
Excessive waiting time in queues	1.11 (0.96–1.27), p = 0.170	1.13 (1.02–1.26), p = 0.020	1.10 (1.01–1.21), p = 0.043
High medical costs	2.30 (2.03–2.62), p < 0.001	1.77 (1.63–1.93), p < 0.001	1.23 (1.15–1.32), p < 0.001
Low medical standards	2.05 (1.82–2.31), p < 0.001	1.70 (1.55–1.87), p < 0.001	1.32 (1.22–1.44), p < 0.001
**Sex (Ref: Male)**			
Female	0.76 (0.61–0.95), p = 0.013	0.87 (0.74–1.01), p = 0.074	1.01 (0.90–1.14), p = 0.872
**Age (Ref: 17–30 years)**			
31–45 years	2.01 (1.18–3.41), p = 0.009	1.88 (1.39–2.54), p < 0.001	1.51 (1.20–1.90), p < 0.001
46–60 years	2.42 (1.41–4.16), p = 0.001	1.80 (1.31–2.47), p < 0.001	1.15 (0.90–1.46), p = 0.251
61–75 years	2.81 (1.59–4.96), p < 0.001	1.61 (1.13–2.28), p = 0.007	1.01 (0.77–1.32), p = 0.942
**Education (Ref: Low)**			
Middle	1.31 (1.02–1.69), p = 0.047	1.62 (1.34–1.95), p < 0.001	1.82 (1.56–2.12), p < 0.001

Variable	Very unfair vs Very fair	Somewhat unfair vs Very fair	Relatively fair vs Very fair
High	1.07 (0.71–1.62), p = 0.859	1.90 (1.45–2.50), p < 0.001	1.82 (1.47–2.26), p < 0.001
**Marital status (Ref: Unmarried)**			
Married	1.55 (0.92–2.63), p = 0.095	1.33 (0.98–1.80), p = 0.068	1.26 (1.00–1.60), p = 0.058
Divorced	2.43 (1.22–4.84), p = 0.012	1.24 (0.77–1.99), p = 0.374	1.07 (0.73–1.57), p = 0.729
Widowed	1.27 (0.63–2.55), p = 0.504	0.79 (0.49–1.28), p = 0.348	0.67 (0.46–0.97), p = 0.035
**Ethnicity (Ref: Han)**			
Other	0.25 (0.16–0.39), p < 0.001	0.28 (0.21–0.37), p < 0.001	0.41 (0.35–0.49), p < 0.001
**Employment (Ref: Employed)**			
Employed but on leave	1.38 (0.76–2.50), p = 0.326	2.07 (1.41–3.03), p < 0.001	1.86 (1.34–2.58), p < 0.001
Unemployed	1.20 (0.93–1.53), p = 0.148	1.26 (1.05–1.52), p = 0.015	1.29 (1.12–1.50), p < 0.001
Student	0.41 (0.15–1.16), p = 0.093	0.50 (0.32–0.77), p = 0.002	0.97 (0.74–1.27), p = 0.825
**Insurance (Ref: No insurance)**			
UEBMI	0.74 (0.51–1.08), p = 0.119	1.08 (0.83–1.41), p = 0.574	1.37 (1.10–1.70), p = 0.004
URBMI	0.77 (0.53–1.14), p = 0.190	1.00 (0.76–1.32), p = 1.000	1.05 (0.84–1.31), p = 0.665
Government-funded	0.44 (0.16–1.20), p = 0.109	0.68 (0.37–1.23), p = 0.204	1.37 (0.90–2.10), p = 0.148

Variable	Very unfair vs Very fair	Somewhat unfair vs Very fair	Relatively fair vs Very fair
NRCMS	0.73 (0.54–0.99), p = 0.044	0.96 (0.76–1.22), p = 0.737	1.08 (0.89–1.30), p = 0.422
Critical Illness	0.41 (0.11–1.50), p = 0.179	0.50 (0.21–1.21), p = 0.125	1.05 (0.61–1.82), p = 0.862

Note: OR = odds ratio; CI = confidence interval. Reference categories are indicated in parentheses. UEBMI = Urban Employee Basic Medical Insurance; URBMI = Urban Resident Basic Medical Insurance; NRCMS = New Rural Cooperative Medical Scheme. The dependent variable is perceived healthcare fairness, with "very fair" as the reference category. p < 0.001 indicates that the exact p-value is below 0.001.

Table S3. Dimensionality analysis of the five healthcare-barrier items.

Item	EFA loading (1-factor)	CFA standardized loading (1-factor)	CFA standardized loading (2-factor)
e3c_1 (distance)	0.624	0.623	0.633 (barrier1)
e3c_2 (appointment wait)	0.895	0.897	0.902 (barrier1)
e3c_3 (queue wait)	0.882	0.871	0.882 (barrier1)
e3c_4 (cost)	0.662	0.702	0.825 (barrier2)
e3c_5 (medical standards)	0.544	0.586	0.654 (barrier2)
**Eigenvalue**	2.73	—	—
**Variance explained**	54.1%	—	—
**CFI**	—	0.992	0.999
**TLI**	—	0.985	0.997
**RMSEA**	—	0.100	0.047
**SRMR**	—	0.048	0.022
**Factor correlation**	—	—	0.792
**Cronbach's α**	0.79	—	—

Note: EFA = exploratory factor analysis with polychoric correlations; CFA = confirmatory factor analysis with WLSMV estimator. In the two-factor model, barrier1 = distance, appointment wait, and queue wait; barrier2 = cost and medical standards.

Table S4. Sensitivity analysis of insurance classification.

Insurance classification	Economic barriers OR (95% CI), somewhat unfair vs very fair	Capacity barriers OR (95% CI), somewhat unfair vs very fair	Consistent with primary analysis?
Primary hierarchy	1.77 (1.63–1.93)***	1.70 (1.55–1.87)***	—
Multiple insurance as separate category	1.77 (1.62–1.92)***	1.70 (1.55–1.86)***	Yes

*Note: OR = odds ratio; CI = confidence interval. ***p* < 0.001. The sensitivity analysis treated multiple insurance types as a separate category (n = 463).*
